# Giant viral signatures on the Greenland ice sheet

**DOI:** 10.1186/s40168-024-01796-y

**Published:** 2024-05-17

**Authors:** Laura Perini, Katie Sipes, Athanasios Zervas, Christopher Bellas, Stefanie Lutz, Mohammad Moniruzzaman, Rey Mourot, Liane G. Benning, Martyn Tranter, Alexandre M. Anesio

**Affiliations:** 1 Department of Environmental Science, Aarhus University, Roskilde, 4000 Denmark; 2 Department of Ecology, Innsbruck University, Innsbruck, 6020 Austria; 3 Department of Agroecology and Environment, Plant-Soil Interactions, Agroscope, Zurich, Switzerland; 4 German Research Centre for Geosciences, Helmholtz Centre Potsdam, Telegrafenberg, Potsdam, 14473 Germany; 5 Department of Biological Sciences, Rosenstiel School of Marine, Atmospheric and Earth Science, University of Miami, Coral Gables, FL USA; 6 Department of Earth Sciences, Freie Universität Berlin, Berlin, 12249 Germany

## Abstract

**Background:**

Dark pigmented snow and glacier ice algae on glaciers and ice sheets contribute to accelerating melt. The biological controls on these algae, particularly the role of viruses, remain poorly understood. Giant viruses, classified under the nucleocytoplasmic large DNA viruses (NCLDV) supergroup (phylum Nucleocytoviricota), are diverse and globally distributed. NCLDVs are known to infect eukaryotic cells in marine and freshwater environments, providing a biological control on the algal population in these ecosystems. However, there is very limited information on the diversity and ecosystem function of NCLDVs in terrestrial icy habitats.

**Results:**

In this study, we investigate for the first time giant viruses and their host connections on ice and snow habitats, such as cryoconite, dark ice, ice core, red and green snow, and genomic assemblies of five cultivated Chlorophyta snow algae. Giant virus marker genes were present in almost all samples; the highest abundances were recovered from red snow and the snow algae genomic assemblies, followed by green snow and dark ice. The variety of active algae and protists in these GrIS habitats containing NCLDV marker genes suggests that infection can occur on a range of eukaryotic hosts. Metagenomic data from red and green snow contained evidence of giant virus metagenome-assembled genomes from the orders Imitervirales, Asfuvirales, and Algavirales.

**Conclusion:**

Our study highlights NCLDV family signatures in snow and ice samples from the Greenland ice sheet. Giant virus metagenome-assembled genomes (GVMAGs) were found in red snow samples, and related NCLDV marker genes were identified for the first time in snow algal culture genomic assemblies; implying a relationship between the NCLDVs and snow algae. Metatranscriptomic viral genes also aligned with metagenomic sequences, suggesting that NCLDVs are an active component of the microbial community and are potential “top-down” controls of the eukaryotic algal and protistan members. This study reveals the unprecedented presence of a diverse community of NCLDVs in a variety of glacial habitats dominated by algae.

**Supplementary Information:**

The online version contains supplementary material available at 10.1186/s40168-024-01796-y.

## Introduction

Snow and glacier ice algae thrive on the surface of glaciers and ice sheets worldwide during the summer melt season [1–12], producing landscape-wide blooms visible on satellite imagery [13]. Red snow patches on the Greenland ice sheet (GrIS) are dominated by *Chloromonas* spp. and *Chlamydomonas* spp. (Chlorophyta), while glacier ice algal blooms are dominated by *Ancylonema alaskanum* and *Ancylonema nordenskioeldii* (Streptophyta) species [2, 14]. These algae belong to different taxonomic groups, but they both decrease the surface albedo of the snow and ice, which in turn accelerates melting [15–20]. Recent extensive efforts to expand knowledge on the ecology, physiology, and phylogeny of these primary producers’ have so far produced relatively little about their life cycle, including the top-down controls that influence their expansion.

Viruses are abundant and ubiquitous across the whole biosphere [21, 22], including cold [23–27] and polar regions [28–35]. Viruses play an essential role in influencing microbial communities through lysis, metabolic reprogramming, and horizontal gene transfer [36, 37]. The viral shunt within aquatic ecosystems significantly influences the structure of algal blooms and eukaryotic communities [38], thereby impacting local, regional, and global biogeochemical cycles [39] and playing a central role in the termination of marine algal blooms [40, 41]. Most of the nucleocytoplasmic large DNA viruses (NCLDV) investigations typically address marine and freshwater environments [42] and only a few from other environments, including polar regions [43].

NCLDVs (Nucleocytoviricota phylum), also called giant viruses, are a supergroup of double-stranded DNA viruses that infect eukaryotes, possessing large virions (up to 1.2 μm in Pithoviridae [44]) and genome sizes (up to 2.5 Mb in Pandoraviridae [45]). They present a set of signature genes used for phylogenetic analyses but encode genes typical of cellular life, such as tRNA and genes involved in protein biosynthesis [46]. The infection strategies of NCLDVs vary considerably, although similarities in how these viruses enter and exit the host cell can be found [47]. NCLDVs are found as free-living particles in environmental samples, and partial or complete viral genomes have been found to be endogenized in several green algae and other hosts genomes [48, 49]. Nucleocytoviricota was revised after the discovery of unclassifiable families, with the addition of new taxonomic ranks, partitioning them into 6 orders (Chitovirales, Asfuvirales, Pimascovirales, Pandoravirales, Algavirales, and Imitervirales), 32 families, and 344 genera [50]. Recently, taxonomic updates were adopted within the order Imitervirales [50]. This level of viral diversity presents challenges when characterizing environmental samples because of the inherent difficulty of culturing virus-host systems. However, diverse environmental metagenomic studies have emphasized their distribution and diversity, demonstrating their presence in oceans, freshwater, and soil [51, 52], as well as extreme habitats, such as the bathypelagic deep sea ocean [27], marine waters, lakes in Antarctica [53–55] and marine waters, cryoconite holes, and an epishelf lake in the Arctic [25, 29, 35].

In this study, we demonstrate through analysis of both metagenomic and metatranscriptomic data that NCLDVs are a key constituent of environmental snow and ice microbial communities from the Greenland ice sheet (GrIS). Habitats analyzed include the following: cryoconite (*n* = 1), ice core (*n* = 3), green snow (*n* = 2), red snow (*n* = 5), and dark ice (*n* = 8), including the analysis of one metavirome (< 0.2-µm fraction) from dark ice samples. Furthermore, we assess NCLDVs endogenization within cultivated snow algae genomic assemblies (Chlorophyta). Environmental samples were evaluated for the presence of 10 NCLDV marker genes, encoding for factors for maturation of the viral capsid (MCPs), packaging ATPase (A32), DNA polymerase elongation subunit family B (PolB), D5-like helicase-primase (D5), mRNA-capping enzyme (mRNAc), RNA polymerase large and small subunit (RNApl, RNAps), DNA or RNA helicases of superfamily II (RNR, SFII) and poxvirus late transcription factor VLTF3 like (VLTF3), and their clustering with known viral families. Retrieval of 10 giant virus metagenome-assembled genomes (GVMAGs) that were assigned to the Imitervirales, Asfuvirales, and Algavirales was undertaken for comparison with metagenomic and metatranscriptomic viral genes present in these environmental samples to assess the potential viral influence on snow and glacier ice algal blooms.

## Results and discussion

**Fig. 1:**
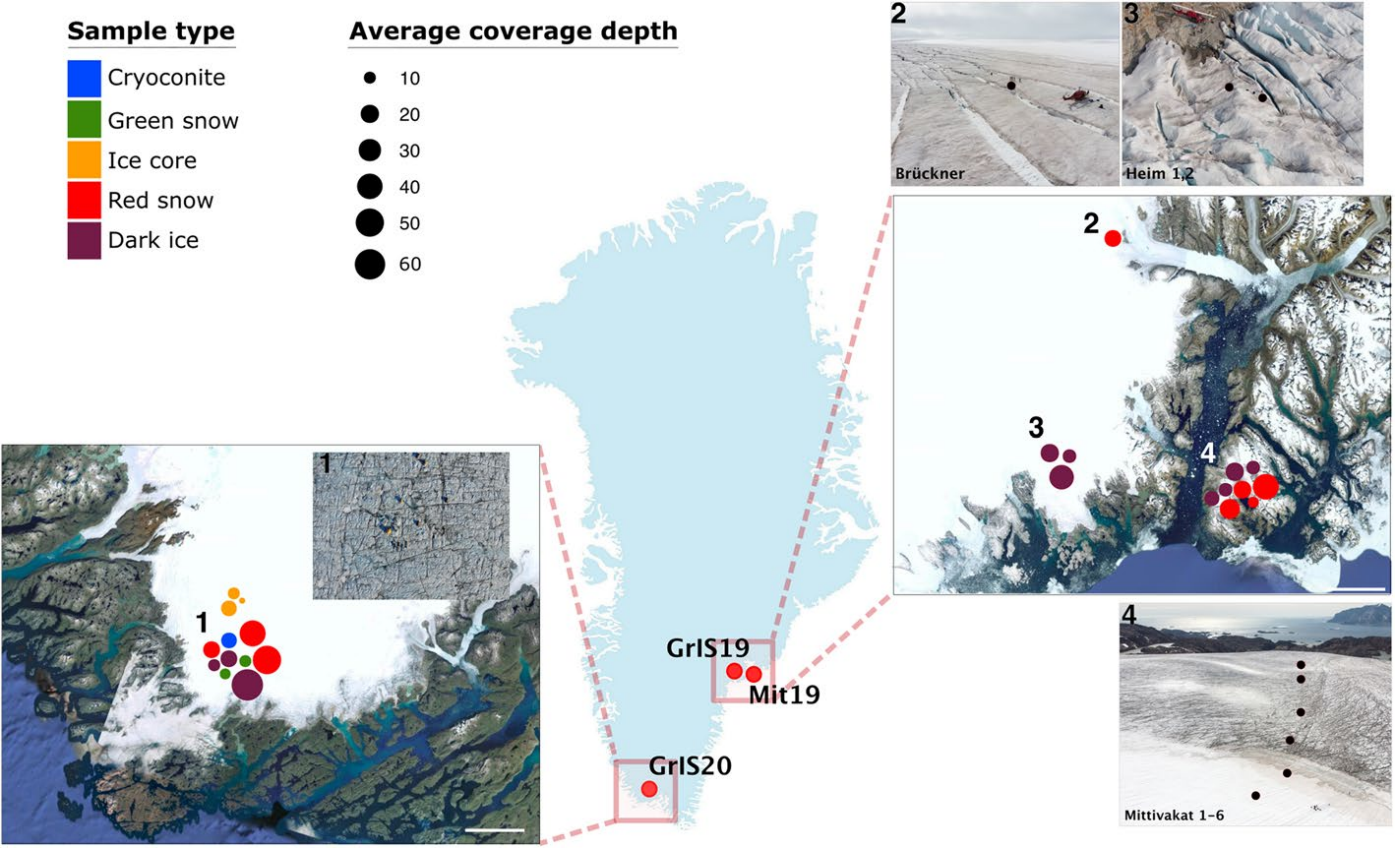
Greenland 2019 and 2020 sampling campaigns (GrIS19/Mit19 and GrIS20, respectively) for environmental samples. One location on the south side of the Greenland ice sheet (inset 1, bottom left). Three locations on the east side of the Greenland ice sheet: Bruckner Glacier (inset 2, top right), Heim Glacier (inset 3, top right), and Mittivakkat Glacier (inset 4, bottom right). Sample types include the following: cryoconite sediment, ice core, dark surface ice, and green and red snow. Circle sizes indicate the metagenome library’s average coverage depth. (Sample information can be found in Supplementary Table 1)

We highlight the unprecedented presence of NCLDV marker genes in microbial communities within Greenland ice sheet surface environments, including cryoconite, dark ice, ice core, and red and green snow, and within the genomic assemblies of five cultivated Chlorophyta snow algae (Fig. 1, Tables S1 and S2).

**Fig. 2:**
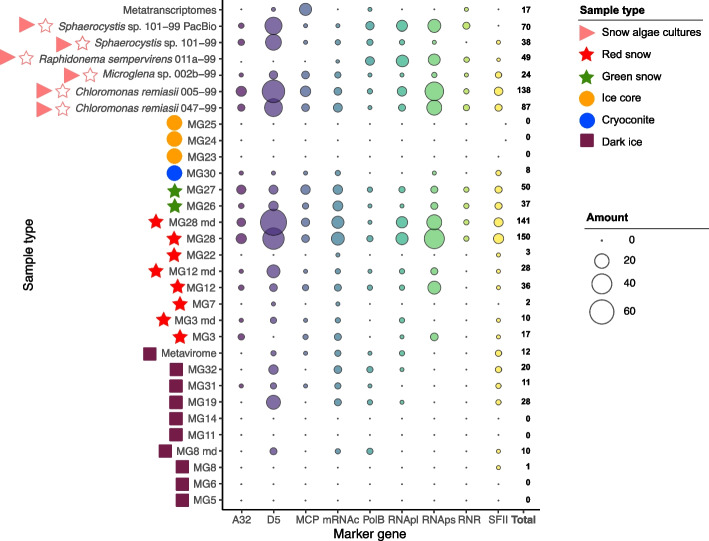
Quality-controlled counts of unfragmented NCLDV marker genes after homology searches against the NCBI nr reference database for each sample of this study. Analysis was carried out in 19 environmental metagenomes (MG) and 18 environmental metatranscriptomes (pooled) obtained from samples of cryoconite (*n* = 1), ice core (*n* = 3), green snow (*n* = 2), red snow (*n* = 5), dark ice (*n* = 8)), 1 metavirome (dark ice), and 5 snow algae genomic assemblies from the CCCryo collection. The points represent the total number of each marker gene in the samples with “total” indicating the overall count of marker genes in that sample. The “md” (more depth) notation following selected samples represents those that were re-sequenced with higher metagenomic coverage. Colored symbols on the left of the sample names represent the sample types

To reduce false positives of NCLDV marker gene identification, fragmented hits were removed and verified against the NCBI nonredundant (nr) database (April 2023), bolstering the quality of the remaining genes used for phylogenetic comparisons. Inconclusive matches occurred with all tested marker genes, mainly due to the presence of hypothetical proteins generated from poorly annotated bacterial MAGs and unknown endogenous viruses within eukaryotic genomes in the database. This resulted in a total of 879 marker genes, 387 from red snow, 298 from snow algae genomic assemblies, 87 from green snow, and 82 from dark ice (Fig. 2, Figs. S1–S7, Tables S2–S4).

To better speculate on the total number of NCLDV’s present, MCP genes were summed and used as a proxy for the number of NCLDVs as they are considered *bona fide* viral genes [50]. Before stringent quality control measures were applied, there were 211 MCP genes; after quality control, only 67 remained. While this stringent quality control method may limit the detection of novel NCLDVs (the original amount of MCP genes was nearly three times higher), it still emphasizes the potential diversity and abundance of the NCLDVs across the Arctic. The 19 environmental metagenome libraries varied in library coverage and size; however, these values did not relate to the number of NCLDV marker genes identified (Fig. 1, Table 1, Tables S1, S2–S4). NCLDV genes were absent in the three ice cores or in four out of nine dark ice metagenomes (Fig. 2, Table S4).

**Table 1:** Environmental sample name, sample type, location, filed campaign and year, nucleic acid extraction method (coextraction with PowerLyzer PowerSoil DNA and RNeasy PowerSoil Total RNA kit (denoted with DNA/RNA) or cetyltrimethyl ammonium bromide (denoted with CTAB), or DNA purification resin (denoted with resin)), sequencing platform, and assembly size in base pairs for each environmental sample. The “md” (more depth) notation following selected samples represents those that were re-sequenced with higher metagenomic coverage

Sample	Sample type	Location	Field campaign and year	Extraction	Sequencer	Total length (Mbp) (MG/MT)	No. of contigs (> = 1000 bp) (MG/MT)
MG11	Dark ice	Mittivakkat	MIT19	DNA/RNA	NextSeq	114/0.046	27,406/14
MG12	Red snow	Mittivakkat	MIT19	DNA/RNA	NextSeq	42/0.101	9889/19
MG12_md	Red snow	Mittivakkat	MIT19	DNA/RNA	NextSeq	53	14,110
MG14	Dark ice	Mittivakkat	MIT19	DNA/RNA	NextSeq	68/0.064	18,419/11
MG19	Dark ice	Heim	GrIS19	DNA/RNA	NextSeq	84/0.136	18,644/44
MG22	Red snow	GrIS	GrIS20	DNA/RNA	NextSeq	68/0.142	14,251/37
MG23	Core	GrIS	GrIS20	DNA/RNA	NextSeq	78/0.037	21,049/5
MG24	Core	GrIS	GrIS20	DNA/RNA	NextSeq	121/0.021	34,539/6
MG25	Core	GrIS	GrIS20	DNA/RNA	NextSeq	87/0.058	22,863/18
MG26	Green snow	GrIS	GrIS20	DNA/RNA	NextSeq	81/0.180	19,578/44
MG27	Biofilm	GrIS	GrIS20	DNA/RNA	NextSeq	57/0.076	13,049/12
MG28	Red snow	GrIS	GrIS20	DNA/RNA	NextSeq	15/0.434	3124/98
MG28_md	Red snow	GrIS	GrIS20	DNA/RNA	NextSeq	32	17,670
MG3	Red snow	Mittivakkat	MIT19	DNA/RNA	NextSeq	51/0.093	11,233/20
MG3_md	Red snow	Mittivakkat	MIT19	DNA/RNA	NextSeq	161	45,184
MG30	Cryoconite	GrIS	GrIS20	DNA/RNA	NextSeq	114/0.014	28,357/4
MG31	Dark ice	GrIS	GrIS20	DNA/RNA	NextSeq	47/0.150	11,766/38
MG5	Dark ice	Mittivakkat	MIT19	DNA/RNA	NextSeq	123/0.076	33,413/19
MG6	Dark ice	Mittivakkat	MIT19	DNA/RNA	NextSeq	72/0.100	19,414/28
MG7	Red snow	Bruckner	GrIS19	DNA/RNA	NextSeq	58/0.123	15,900/30
MG8	Dark ice	Heim	GrIS19	DNA/RNA	NextSeq	72/0.060	18,115/12
MG8_md	Dark ice	Heim	GrIS19	DNA/RNA	NextSeq	114/0.046	40,113
MG32	Dark ice	GrIS	GrIS20	DNA (CTAB)	Nanopore MinION	42/0.101	3454
Metavirome	Dark ice	GrIS	GrIS20	DNA (Resin)	NextSeq	53	25,149

*GrIS* stands for Greenland ice sheet, *MG* stands for metagenome, *MT* stands for metatranscriptome

**Fig. 3:**
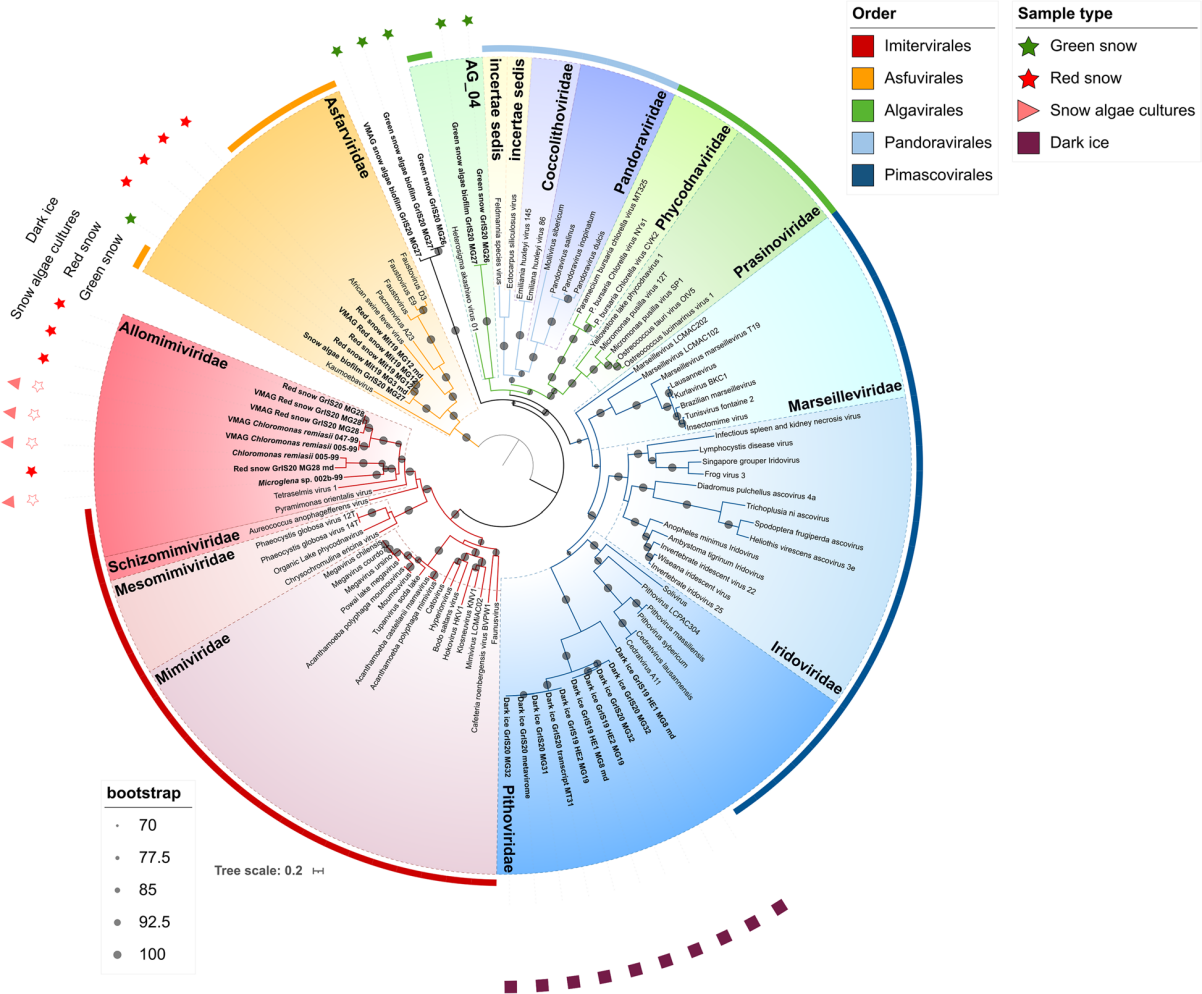
Maximum-likelihood phylogenetic tree of the NCLDV core gene DNA polymerase (PolB). Sequences recovered from the environmental samples are presented in bold at the tree node. Environmental sample types are specified in correspondence of each sequence. Branches are color-coded by order-level taxonomy. Viral families are specified in the colored ranges. The dark dots at the nodes represent the bootstrap support value of > 70

The D5, RNApl, and RNAps marker genes were the most abundant in all metagenome samples making up > 50% of the marker genes (Fig. 2, Figs. S1–S3, Tables S2–S3). Similarly, RNApl and RNAps genes in the pooled metatranscriptomes had the most individual counts (35% and 32%, respectively) of the transcribed gene (Tables S2–S3; *e*-value = 1 × 10^−10^). However, none of these transcribed sequences matched known NCLDV members on NCBI nr and was excluded from further analyses. In most cases, the signatures of these single NCLDVs marker genes found both in metagenomic and metatranscriptomic data were found in short contigs, impeding a deeper investigation of genomic context. Transcribed MCP genes were the third most abundant (18%) in the metatranscriptomes (Figs. S4 and S8, Tables S2–S4) after RNApl and RNAps counts, confirming high expression in the environment [56]. All the sequences of the transcribed MCP genes were similar to known NCLDV families and clustered closely to the related metagenomic sequence within the phylogenetic tree (Fig. S4). Generally, the marker gene sequences were closely related and clustered on shared phylogenetic tree nodes, despite originating from different metagenomes or genomes. For example, MCP genes from red snow samples, MG12 and MG3, were > 97% identical (Fig. S5) and from an environmental red snow sample (MG28) and *Chloromonas remiasii* 005–99 or 047–99 were up to 84% identical. Furthermore, the dark ice samples, MG32, MG31, MG19, MG8, and the metavirome, each contained a PolB sequence with a high percentage of identity, > 99.5% (Fig. 3, Table S5).

**Fig. 4:**
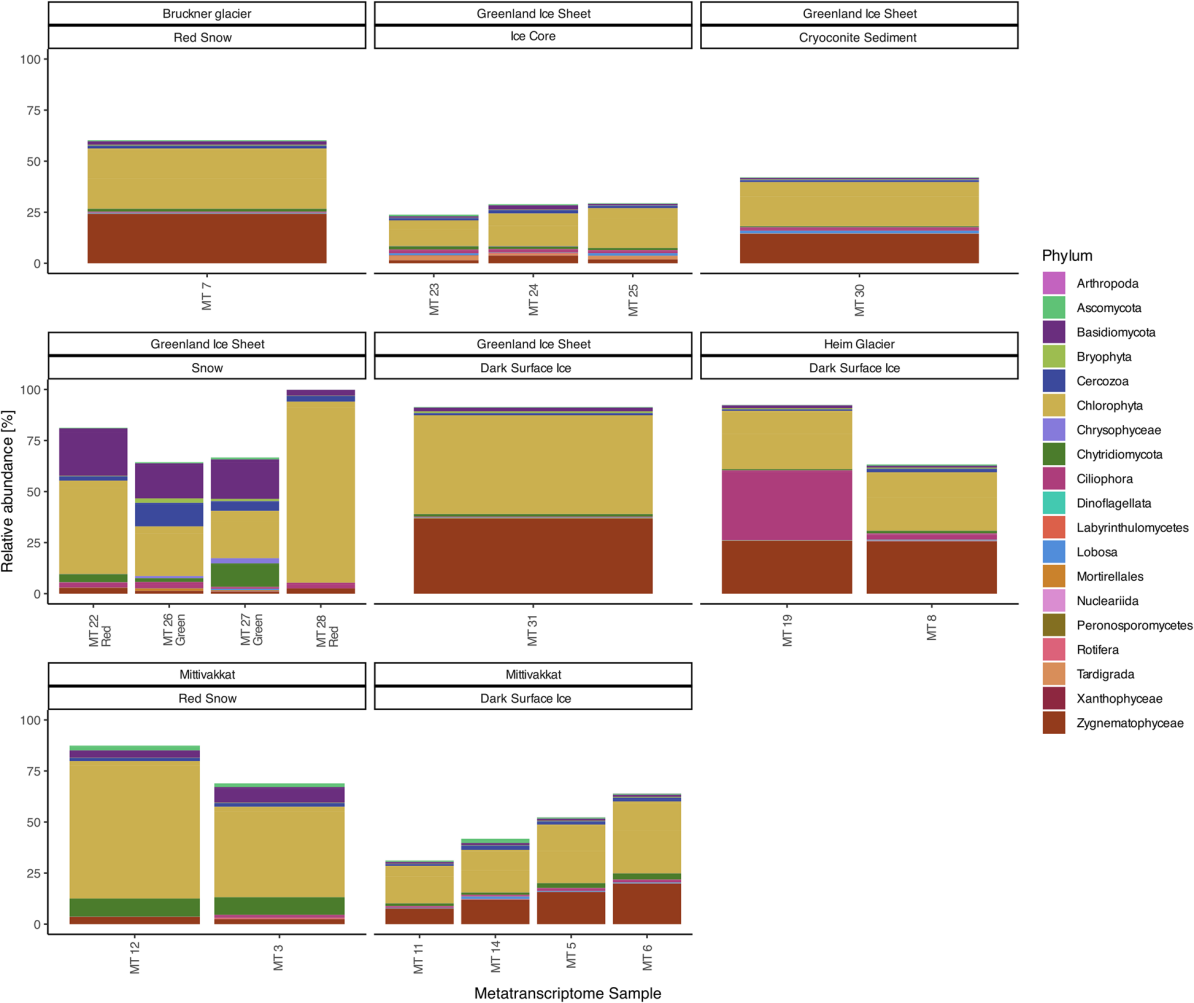
18S rRNA diversity of the 18 environmental samples from TotalRNA. Some eukaryotic phyla are made up of more than one individual. Bacterial phyla are not displayed but make up the empty space above each bar. Calculated relative abundance percentages can be found in Supplementary Table S9

Red snow samples MG12 and MG3 from the GrIS contained PolB genes that were > 99.4% identical (Fig. 3). The mRNAc genes from dark ice samples, MG32, MG31, MG19, and the metavirome, were > 99.7% identical (Fig. S6). This gene similarity suggests a degree of relatedness with the NCLDVs identified at each environment, despite unique sample types. The eukaryotic diversity and composition in each of the locations are generally composed of the same members (Figs. 4 and S9), which corroborates identifying similar NCLDVs marker genes.

Additionally, D5 marker genes from red snow samples and two of the algae genomic assemblies clustered closely with giant endogenous viral elements (GEVEs) previously found in diverse green algae genomes [48] (Fig. S1). The co-clustering of marker genes found in snow algae genomic assemblies with those from environmental red snow samples (Fig. 3, S1–S7) and GEVEs observed in other green algae (Fig. S1) strongly suggests that chlorophytes may serve as hosts in this environment and have endogenized viral genes.

In total, 10 GVMAGs (Table S6) and 29 individual PolB sequences (Table S4) were retrieved from the 31 different samples investigated, 7 GVMAGs and 22 PolB originating from the environment, and 3 GVMAGs and 7 PolB from the snow algal genome assemblies (Fig. 2, Table S4). Since PolB is the only marker gene typically found as single copy, it is used for phylogenetic placement within known NCLDV families [57]. In red snow and dark ice samples, a few PolB sequences had similar or identical residues (Table S5). Between the 10 GVMAGs, there were 8 unique genome pairs with > 99.1% ANI (Table S7). The origin of these identical GVMAGs were red snow samples from Mittivakkat Glacier (MG12 and MG3) and two *Chloromonas remiasii* cultures (005–99 and 047–99), further showing giant virus links to red snow algae. However, the GVMAGs retrieved here are not an exhaustive representation of the NCLDVs present in these Greenland environments. There were five MAGs that had less than five NCLDV marker genes, smaller than 100-kbp genome size, and therefore were not considered further as GVMAG. One was from the cryoconite sample (MG30), one from green snow (MG27), two from red snow (MG3_md and MG12), and one from dark ice (MG32). Although these are poor representative, they still indicate potential GVMAG diversity in other habitats in the GrIS. The functional annotation of the 10 GVMAGs highlighted the presence of genes associated with eukaryotic photosynthesis, such as heliorhodopsin, Rubisco LSMT substrate binding, bestrophin chloride channel, and copper amine oxidase [42]. These annotations were found within one Algavirales and three Asfuvirales GVMAGs (MG12_md_6, MG3_12, MG12_md_5, and MG12_2, respectively) (Table S8). These genes are often found endogenized in host genomes, and finding these within GVMAGs from environmental snow samples further indicates a potential host-viral relationship.

Individual phylogenetic trees of marker genes were built to examine the phylogenetic relationships (PolB, Fig. 3) and phylogenetic diversity [50] (D5, RNAps, RNApl, MCP, mRNAc, A32, SFII, VLTF3, and RNR, Figs. S1–S7) between the proteins found in the metagenomic, metatranscriptomic, metaviromic, and genomic contigs in comparison with known viruses. The maximum-likelihood phylogenetic tree of the NCLDV marker gene DNA polymerase (PolB) showed clustering with four known viral families (Allomimiviridae, Pithoviridae, Asfarviridae, Algavirales AG-04), with a clear separation in terms of NCLDV groups based on the sample type (Fig. 3). PolB sequences from red snow samples (three identified in MG12 and one in MG3) and green snow samples (1 sequence from MG27) grouped together with Asfuvirales reference sequences, which is a globally distributed group in the ocean known to infect photosynthetic dinoflagellates, as well as protozoans [58]. The rest of the red snow (MG28, four sequences) and sequences originating from the snow algae genomic assemblies (*C. remiasii*, 3 sequences, and *Microglena* cf. sp., 1 sequence) clustered with the Imitervirales, which is the widest order infecting a variety of hosts, including green algae [52, 59]. Signatures found in green snow samples (one sequence retrieved from MG27 and one from MG26) formed a sister group with the *Heterosigma akashiwo* virus 01 (Algavirales), which has been used as microbiological agent for red tide control in the ocean [60]. Sequences from dark ice were assigned to Pithoviridae (two sequences from MG8, two from MG19, one from MG31, one from MT31, and three from MG32), which mostly infect species of the amoebozoan genus *Acanthamoeba* [47]. Overall, PolB phylogeny shows a wide diversity of NCLDVs and reveals the potential top-down interactions affecting a diverse eukaryotic host community (algae and protists) on the GrIS.

**Fig. 5:**
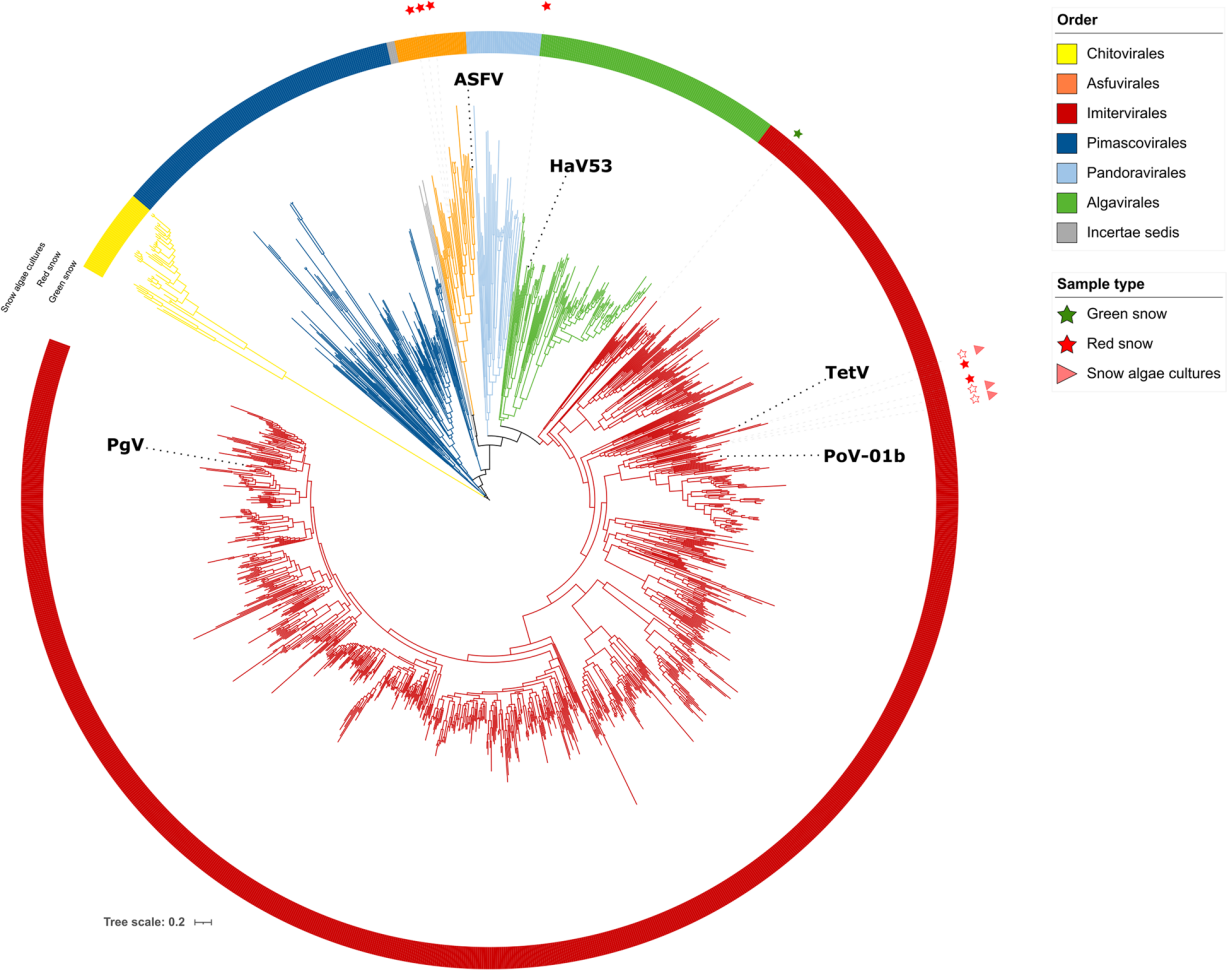
Maximum-likelihood parsimony phylogenetic tree with 1171 external genomes from previously published GVMAGs and 10 GVMAGs from this study. According to the tree, the retrieved GVMAGs cluster in correspondence of the Asfuvirales (3), Imitervirales (6), and Algavirales (1) orders. VGMAGs originated from this study are highlighted by the corresponding environmental sample type symbol. Branches are color-coded by order-level taxonomy. Cultured isolate virus references of interest are labeled in their approximate location along the branches with the following abbreviations: African swine fever virus (ASFV), *Heterosigma akashiwo* Virus 01 isolate HaV53 (HaV53), *Tetraselmis* Virus (TetV), *Pyramimonas orientalis* Virus 01b (PoV-01b), *Phaeocystis globosa* Virus (PgV)

Different samples of red snow (MG28, MG12, and MG3) contained NCLDV signatures belonging to different families. The concatenated maximum-likelihood tree assigned all the GVMAGs generated from the GrIS2020 red snow sample (MG28), together with the snow algae *C. remiasii* GVMAGs (Fig. 5), to the family Allomimiviridae, confirming results obtained through PolB phylogeny.

This family contains the recently cultivated *Oceanusvirus kaneohense* [61], formerly known as *Tetraselmis* Virus (TetV), which infects the marine green algae *Tetraselmis* (Chlorodendrophyceae) [62]. Members of this genus are ubiquitous and commonly found in nutrient-rich marine and fresh waters, although the first TetV-specific host was initially isolated from an oligotrophic habitat (open ocean) [62]. GVMAG_MG28_md_2 and *Chloromonas*_*remiasii*_005-99_3 fell within the cluster formed by TetV. Another member of the Allomimiviridae family is the species *Heliosvirus raunefjordenense*, formerly known as *Pyramimonas orientalis* Virus 01b (PoV-01b), also infecting chlorophytes [63]. One GVMAG retrieved from green snow (MG27) was assigned to the family IM_18 of the Imitervirales order. This family is represented only by genomes derived from cultivation-independent approaches retrieved from freshwater and marine sources and does not include isolated members at present. One GVMAG originated from red snow (Mittivakkat 2019) was assigned to the order Algavirales (family *incertae sedis*), an NCLDV order encompassing several well-studied algal viruses [50]. Three GVMAGs originating from red snow sampled from the Mittivakkat Glacier in SE Greenland were assigned to the Asfuvirales family AF_2, and one was assigned to a cluster with uncertain taxonomy. Generally, members of the Asfuvirales infect a mixture of metazoan and protist hosts and are broadly distributed in marine systems [58, 64]. The presence of Pithoviridae signatures in dark ice and their likely associations with protists suggest that the GVMAGs from red snow assigned to the Asfuvirales are also probably associated with protists hosts. The unassigned GVMAG emphasizes the complexity of NCLDV taxonomy, which is constantly growing from metagenomic data but unfortunately lacks additional cultured isolate reference genomes.

The active 18S rRNA eukaryotic community contained algal and protistan members. Dark ice was dominated by the phylum Streptophyta, mainly from the class Zygnematophyceae (7–37% throughout the seven dark ice samples), but also with the presence of chlorophytes, specifically from two classes, Chlorophyceae (5–21%) and Trebouxiophyceae (9–27%) (Fig. 4 and Tables S9–S10). One dark ice sample was used in an attempt to sequence and assemble a draft genome of the Streptophyta glacier ice algae. The final assembly had more than Streptophyta contigs (Table S11), so it was considered as the 19th environmental sample (MG32, Fig. 4) despite different extraction, sequencing, and assembly methods used on the other metagenomic samples. Green and red snow were dominated by algae belonging to the phylum Chlorophyta (20–22% and 28–69%, respectively). The active protistan community included the cercozoa Glissomonadida and ciliate *Stokesia*, which are commonly found in glaciers, snow, and sea ice [12, 65–67]. The order Glissomonadida includes biflagellate gliding bacterivores and algivorous amoeboflagellates [68, 69], which were mainly present in green (4–10%) and red (1–2%) snow samples (Table S6) but also in dark ice (0–1%) and ice core (1%). *Stokesia* is a large (more than 100 μm) ciliate containing endosymbiotic green algae commonly found within spring phytoplankton blooms in oligo-mesotrophic lakes [70, 71], which was present and active in a dark ice sample from Heim Glacier (MG19, 33%, Table S6). The variety of active algae and protists in these GrIS habitats containing NCLDV marker genes suggests that infection can occur on a range of eukaryotic hosts.

The presence of active members of the community in all sample types was confirmed by the read recruitment analysis showing that reads of the metagenomic samples recruit to the corresponding metatranscriptomic sample. Most of the reads from each sample mainly mapped to their respective assemblies, however also mapped to other environmental types (10 GVMAGS, 23 metagenomes, 1 metavirome, 18 metatranscriptomes (Table S12, Fig. S10)). For example, the Streptophyta-dominated environmental sample (MG32, Fig. S9) mapped 30% of the reads to its own assembly, 4% mapping to red snow or dark ice assemblies, and 8% mapping to the cryoconite assembly (Fig. S10, Table S12). Furthermore, the red snow sample MG28 mainly mapped (27% reads) to a metatranscriptome assembly from green snow (MG27, Fig. S10). This pattern, where one sample type (e.g., red snow) maps at least 5% of the reads to another sample type assembly (e.g., green snow), demonstrates the community overlap between Greenland ice sheet habitat types. This is further seen within the hierarchical clustering groups through the shared read recruitment analysis, where different sample types share similar read recruitment pattern (Fig. S10). The similarities in shared mapping are better underscored by the compared diversity of the 18S rRNA from the metagenomes and metatranscriptomes (Fig. S11). These glacial samples observed diversity was above 325 in most metatranscriptomic samples, except 2 red snow samples (MT12 and MT28, Fig. S11A, Table S1). These two red snow samples, and the deeply sequence MG28, also have a lower observed diversity in 18S rRNA genes from the metagenomes (Fig. S11C). The Shannon index highlighted a high diversity in the samples (Fig. S11A), excluding few exceptions such as the red snow samples MT28, MT12, and MT22 that seemed to harbor a lower diversity (2, 2.9, and 3.3, respectively) and two dark ice samples MT19 and MT31 (2.9, and 2.6, respectively). Overall, the inverse Simpson index showed a low evenness of the samples (ranging between 5.2 and 47) that therefore appeared to be dominated by few taxa (Fig. S11A). The nonmetric multidimensional scaling (NMDS) analysis revealed clustering of the dark ice, ice core, and red snow samples based on the location over a strong association with sample type for both metagenome and metatranscriptomic samples (Figs. S11B and D). Generally, samples also clustered based on sample type, with the exception of the red snow sample MG28, which appeared significantly dissimilar from the others (Fig. S11B).

Overall, metagenomic evidence reveals diverse NCLDV signature genes in snow and ice habitats, highlighting the presence of potential viral controls on these algal communities. Furthermore, the presence of viral genes in *Chloromonas* spp., *Microglena* sp., and *Sphaerocystis* sp. genomic assemblies can be most likely considered a result of past viral DNA integration, as seen already in other non-snow hosted green algae (Chlorophyta) [48] and highlighted by genomic evidence in a comprehensive survey of giant virus DNA integration into genomes of algae and protists [49]. Integration of endogenous viruses in algal genomes is not present in all algal groups and appears to be highly host specific [49]. Nevertheless, the presence of endogenized viruses does impacts the algal genome evolution and potentially the ecological success of these algae [47]. Viruses would not be endogenized in the first place without active viral-host interactions. The co-clustering of metagenomic and endogenized signatures on the phylogenetic trees indicates that these NCDLVs are likely close relatives and allows the host to be inferred. It suggests that the Allomimiviridae group of NCLDV signature genes in these red snow samples is from algal-infecting viruses.

These diverse environmental sample types offer valuable insights into the prevalence of NCLDVs within microbial communities on the Greenland ice sheet. They are primarily associated with snow algae (Chlorophyceae) in red snow, while other signatures, such as Asfarviridae and Pithoviridae, are linked to protists in dark glacier ice algae-dominated habitats. Collectively, these findings suggest that pigmented supraglacial snow and ice habitats contain a diverse array of NCLDVs linked to various eukaryotic hosts. Furthermore, the detection of transcribed viral marker genes that taxonomically identify with NCLDV metagenomic sequences implies an active NCLDV influence on the snow and ice algal community, potentially serving as regulators of colored snow blooms.

## Material and methods

### Samples collection and preparation

Samples were collected during two fieldwork campaigns in July 2019 and July 2020. Samples in 2019 were collected from three locations in the SE of the GrIS. Mittivakkat Glacier is an independent glacier separated from the GrIS, located on Ammassalik Island, in South-East Greenland, below the Arctic Circle (65.69°N; 37.83°W) (Fig. 1). The samples from Mittivakkat Glacier were collected along a west-sloping transect from the accumulation zone (two red snow samples) to the ablation area (four dark ice samples). The second location was on the GrIS, across the fjord from Mittivakkat Glacier, and one sample of red snow and two dark ice samples were collected from Bruckner and Heim glaciers (65.99°N; 38.44°W, and 65.95°N; 38.53°W, respectively).

In 2020, a set of environmental samples were collected from the GrIS, close to the QAS_U and QAS_M PROMICE stations (∼61.08°N; 46.83°W and 61.18°N; 46.82°W) in S-Greenland. The samples included ice core (1-m core that included snow and ice transition section, *n* = 3), cryoconite hole sediment (*n* = 1), dark ice (*n* = 1), dark ice sample only for viral fractionation concentration (*n* = 1) and for the purpose of creating a draft genome of the *Ancylonema* ice algae (*n* = 1), green snow algae biofilm (*n* = 1), green snow (*n* = 1), and red snow ((*n* = 2; Fig. 1). Coordinates and details for each sampling site and sample are reported in Table 1 and supplementary material (Table S1). Dark ice represents glacial surface ice that is visually dark as compared to white ice and contains a high abundance (10^4^ cell/ml) of dark pigmented glacier algae, typically dominated by the class Zygnematophyceae [5]. Green and red snow also is visually colored and contains a high abundance of green and red snow algae, both within the class Chlorophyceae [5].

All samples were collected with sterile nitrile gloves and tools and stored in sterile Whirl–Pak® bags. Samples were melted at ambient temperature in the field and filtered through 0.22-μm mixed cellulose ester membrane filters (Sartorius, Germany), which were immediately frozen and transported to the home laboratory in a cryo-shipper at liquid nitrogen temperatures, where they were stored at − 80 °C until further processing. Total DNA and RNA were extracted from the filters with the PowerLyzer PowerSoil DNA isolation kit and the RNeasy PowerSoil Total RNA kit (Qiagen, Germany), respectively, following the manufacturer’s instructions. Nineteen DNA libraries were generated with the NEBNext® Ultra™ II FS DNA Library Prep Kit (Illumina), with 8 rounds of PCR amplification. RNA samples were treated with the DNase Max® kit to remove remaining DNA (Qiagen, Germany) following the manufacturer’s instructions. Eighteen RNA libraries were prepared with the TotalRNA NEBNext® Ultra™ II RNA Library Kit, with 8 rounds of PCR amplification. Sequencing was performed in-house using the NextSeq 500 platform and the 300 cycle v2.5 chemistry (151-bp pair-end reads). The reconstructed, full-length rRNA small subunit (SSU) genes in the 18 environmental transcripts were taxonomically identified with Silva 138.1, BLAST, and CREST4 [72] as part of our in-house TotalRNA workflow (DOI: 10.5281/zenodo.7656004). Chloroplast and mitochondria sequences made up a total of 0.4% to 19.9% of initial sequences and were removed from further analysis. Statistical comparisons of the assembly diversity were analyzed with phyloseq (v 1.44) [73] in R Studio (v 3.17) [74].

### Snow algal genomic amplification and assembly

The algal strains *Microglena* cf. sp. 002b-99, *Chloromonas remiasii* 047–99 and 005–99, cf. *Sphaerocystis* sp. 101–99, and *Raphidonema sempervirens* 011a-99, commonly present on pigmented snow and surface ice of the GrIS and other glaciers, were obtained from the Culture Collection of Cryophilic Algae (CCCryo) at the Fraunhofer IZI-BB Institute (Table 2).

**Table 2:** Snow algae cultures sequenced and assembled from the CCCryo Culture Collection, all extracted with PowerSoil DNA Isolation Kit for DNA sequencing

Culture name	CCCryo code	Sequencer	Total length (Mbp)	No. of contigs (> = 1000 bp)	GC (%)
*Chloromonas remiasii*	047–99	MiSeq	346	48,150	58.8
*Chloromonas remiasii*	005–99	MiSeq	398	74,719	58.8
*Microglena* cf. sp.	002b-99	MiSeq	354	56,785	46.8
*Raphidonema sempervirens*	011a-99	MiSeq	54	9126	49
*Sphaerocystis* sp.	101–99	MiSeq/PacBio	115/102	8784/359	52.5/52.7

They were grown at 10 °C in liquid triple-concentrated Bold’s Basal Medium [75] (pH 5.5) under axenic conditions and continuous illumination as per the CCCryo guidelines. DNA was extracted using the PowerSoil DNA Isolation kit (QIAGEN, Germany) following the manufacturer’s instructions. Each strain was sequenced on a MiSeq flowcell using the 500 cycles v2 chemistry (250-bp pair-end reads) at the Genome Analysis Centre (Earlham Institute, UK). In addition, high-molecular-weight DNA of *Sphaerocystis* sp. 101–99 was extracted using the QIAGEN genomic-tips extraction kit and sequenced using one PacBio Sequel SMRT Cell (2.0 chemistry) at NERC Biomolecular Analysis Facility — Liverpool. The genomes of all five strains were de novo assembled by the Earlham Institute using the Illumina 250-bp paired-end reads. Quality control of the raw data was done using FastQC (fastqc-0.11.2, http://www.bioinformatics.babraham.ac.uk/projects/fastqc/). Preprocessing of the raw reads was done by the Earlham Institute (https://github.com/TGAC/kontaminant) using the pipeline Kontaminant. ABySS (v.1.9.0) [76] was used to perform the de novo assembly of each strain. The PacBio Sequel reads of the strain 101–99 were de novo assembled using Flye (v.2.3.3) [77] using a minimum subread length of 5000 bp and an estimated genome size of 120 Mb. Sample MG32 was extracted with the CTAB (cetyltrimethyl ammonium bromide) method [78] and sequenced on two platforms. Illumina libraries were prepared using the NEBNext® Ultra™ II FS DNA Library Prep Kit (New England Biolabs) and sequenced on a NextSeq 500 instrument with the 300 cycles v2.5 chemistry. Nanopore libraries were prepared using the Ligation Sequencing Kit (LSK-109) and sequenced on a MinION (Oxford Nanopore Technologies, Oxford, UK) with a FLO-MIN106 flow cell, controlled using MinKNOW (19.10.1). Raw nanopore fast5 reads were basecalled with GPU Guppy (3.2.6 + afc8e14). The Illumina reads were quality filtered using trim-galore under default settings (https://github.com/FelixKrueger/TrimGalore). The raw nanopore reads were corrected with the trimmed Illumina reads using LoRDEC [79] with default settings. The corrected long reads were used for de novo whole genome assemblies with Flye [77] under default settings utilizing the − nano-corr flag [77]. under default settings utilizing the − nano-corr flag. The dark ice environmental sample (MG32) containing a high abundance (10^4^ cell/ml) of *Ancylonema* sp. was taken in an attempt of producing a draft genome of the Streptophyta glacier ice algae. The overall appearance of the sample under the microscope gave the misleading impression that this mixed culture would contain primarily *Ancylonema* sp. and prokaryotes. The idea was then to remove prokaryotic contigs and have a representative *Ancylonema* genome. Further analysis on the resulting assembly with BARRNAP (BAsic Rapid Ribosomal RNA Predictor, https://github.com/tseemann/barrnap) revealed the presence of a diverse eukaryotic community. Nevertheless, the sample was kept in the study as it provided another dark ice environmental sample and gave nanopore long reads.

### Metagenome, metatranscriptome, and GVMAG assembly

Illumina reads were quality filtered to remove low-quality reads and trimmed with fastp [80] (version 0.20.0) using default options. Trimmed Illumina reads were assembled with metaSPAdes [81] (v3.15.1) specifying the –only-assembler pipeline. Metatranscriptome reads were quality cleaned and trimmed with trim-galore (https://www.bioinformatics.babraham.ac.uk, v0.6.6) using default options. Raw reads were assembled both singularly (each sample) and pooled together (co-assembly) with Trinity assembler [82] (v2.6.6) including the following options: –normalize_by_read_set. Results of the co-assembly are presented as metatranscriptome — pooled.

Giant virus metagenome-assembled genomes (GVMAGs) were created by binning contigs with MetaBAT2 (v2.12.1) [83] using >  = 5000 base-pair contigs. Resulting bins were analyzed for NCLDV marker genes using ViralRecall (v2) [57] and were considered a GVMAG if they had five or more of the marker genes, a genome larger than 100 kbp, and taxonomic placement within other NCLDV genomes [51]. CoverM (v 0.6.1) (https://github.com/wwood/CoverM) was used to assess the read recruitment between all 57 generated assemblies and the environmental sample reads (18 metagenomes, 19 metatranscriptomes, and 1 metavirome). GVMAGs functional annotations were assessed with InterPro [84] and GVOGs [50].

### Metavirome construction

Five liters of dark ice from the GrIS 2020 location was prefiltered with 3-μm nitrocellulose membrane filters (Sartorius) to remove large particles and subsequently filtered through 0.2-μm VacuCap™ devices (Pall Corporation), retaining the viral fraction (< 0.2 μm). Viruses were further concentrated from the filtrate using iron chloride flocculation [85] followed by storage at 4 °C. After resuspension in ascorbic-EDTA buffer (0.1-M EDTA, 0.2-M MgCl_2_, 0.2-M ascorbic acid, pH 6.0), viral particles were concentrated using Amicon Ultra 100-kD centrifugal devices (Millipore) and extracted as previously described [86]. Briefly, viral particle suspensions were treated with Wizard Polymerase Chain Reaction Preps DNA Purification Resin (Promega, Fitchburg, WI, USA) at a ratio of 1-mL sample to 1-mL resin and eluted with TE buffer (10-mM Tris, pH 7.5, 1-mM EDTA) using Wizard Minicolumns. The DNA library was prepared following the NEBNext® Ultra™ II FS DNA Library Prep Kit (Illumina), with 8 rounds of PCR amplification. Sequencing was performed in-house using the NextSeq 500 platform and the 300 cycle v2.5 chemistry (151 -bp pair-end reads). This sample was processed with a small filter size (< 0.2 µm) and treated as the environmental viral fraction. It is important to note that the small filter size will decrease the amount of NCLDV signatures retrieved.

### Identification of NCLDVs signatures in metagenomic data

ViralRecall was used to identify NCLDV-like sequences and viral-like regions in all the metagenome, metatranscriptomes, metavirome, and pure algal culture. Options used were as follows: -db marker -c. The “marker” option was used to only search against 10 NCLDV marker genes, encoding for factors for maturation of the viral capsid (MCPs), packaging ATPase (A32), DNA polymerase elongation subunit family B (PolB), D5-like helicase-primase (D5), mRNA-capping enzyme (mRNAc), RNA polymerase large and small subunit (RNApl, RNAps), DNA or RNA helicases of superfamily II (RNR, SFII), and poxvirus late transcription factor VLTF3 like (VLTF3). All resulting hits with an *e*-value less than e^-10 were used further. These genes are universal NCLDV marker genes and hence are routinely assessed for identification of signatures of NCLDVs in different ecosystems [51]. PolB is the only marker gene typically found as single copy and is therefore used for phylogenetic placement within known NCLDV families [57].

To confirm that virus-like regions belonged to NCLDV families, blastp function against NCBI nr was used, and 50 top hits were verified for each sequence classified as possible NCLDV gene by ViralRecall. A gene was considered from NCLDV when it had NCLDV results within the top 10 hits. The total abundance of the 10 NCLDV core genes in each sample was calculated before and after verification with NCBI nr by summating the marker genes with an *e*-value cutoff of 1 × 10^−10^ and normalizing to the total library size. Four of the 19 environmental samples with the highest relative presence of viral marker genes (MG3, MG8, MG12, and MG28; Fig. 2) were chosen to be re-sequenced at a greater depth to provide higher sequencing coverage and increase the chances of assembling GVMAGs.

### Phylogeny of unbinned GV marker genes and transcriptomes

MAFFT [87] (v7.475) was used to align the viral regions from sequenced data against the reference sequences using the − auto option to select the appropriate option (L-INS-I, FFT-NS-2, or FFT-NS-i) for each alignment according to the size of input data (options: –maxiterate 1000). Only sequences of marker genes that had an *e*-value <  = 1 × 10^−10^ and had a length comparable to the reference sequences (> = 300 aa) were subsequently kept in the tree. Fragmented signatures (< 300 aa) were not included in the phylogenetic placement. For each gene, maximum likelihood phylogenetic trees were built using IQ-TREE [88] v2.0.3. According to BIC scores, LG + F + I + G4 (PolB) was the best model by the “-m TEST” ModelFinder option [89]. IQ-TREE was run with 1000 ultrafast bootstraps (-alrt 1000 -B 1000) to assess confidence [90].

### Phylogeny of the GVMAGs against 1171 external Nucleocytoviricota genomes

External Nucleocytoviricota genomes were downloaded from previously published studies [50, 91]. All 1171 external genomes and our 10 GVMAGs were aligned using ncldv_markersearch.py (last update 21Apr2022, github.com/faylward/ncldv_markersearch). A maximum-likelihood phylogenetic tree was constructed using IQ-TREE with the LG + F + I + G4 model with -B ultrafast 1000 bootstraps [90]. Phylogeny assignment was assigned based on previous literature [51].

### Supplementary Information

Additional file 1. Figure S1-S7: Maximum-likelihood phylogenetic tree of the NCLDV core gene D5, RNAps, RNApl, MCP, mRNAc, A32, SFII, VLTF3, RNR. Sequences recovered from the environmental samples are presented in bold. Environmental sample types are specified in correspondence of each sequence. Branches are color-coded by order-level taxonomy. Dark dots at the nodes represent the bootstrap support value of >70. Figure S8: Normalized counts of the NCLDV marker genes by each assembly size for all the samples in this study. Analysis was carried out in 19 environmental metagenomes (MG) and 18 environmental metatranscriptomes (pooled) obtained from samples of cryoconite (n=1), ice core (n=3), green snow (n=2), red snow (n=5), dark ice (n=8), one metavirome (dark ice) and five snow algae culture genomic assemblies from the CCCryo collection. The ‘md’ (more depth) notation following select samples are those that were re-sequenced with higher metagenomic coverage. Symbols represent the sample types. Figure S9: Metagenomic SSU relative abundance. Sample descriptions can be found in Table 1 and Supplementary Table 1. The blank space above each bar is comprised of bacterial phyla. The ‘md’ (more depth) following a sample name marks those that were sequenced with a high average library coverage. The full abundance table can be found in Supplementary Table S10. Figure S10: Read mapping percentage transformed into log scale + 0.01 for comparable scales. Purple values are 0 reads mapped. Assemblies (left) and sample read files (right), MG are metagenomes, MG with ‘_2’ note the four samples that were sequenced with higher library coverage, and ‘MT’ are metatranscriptomes. The only culture to have reads recruit was Raphidonema_sempervirens_LIV13260. Sample types are labeled in the same way as Fig. 2.

Additional file 2. Table S1: Samples info. Table S2: ViralRecall GV Count. Table S3: Normalized VRGV Counts. Table S4: NCBI QC GV Counts. Table S5: % identity PolB. Table S6: GVMAG. Table S7: GVMAG ANI %. Table S8: GV MAGs funct ann. Table S9: Percentages 18S rRNA MT. Table S10: Abun Table MetagenomeSSU. Table S11: BARRNAP results. Table S12: CoverM-Mapping

Additional file 3.

## Data Availability

All metagenomes, metatranscriptomes, and culture genomic assemblies can be found under NCBI BioProject: PRJNA1011216 and BioSamples within. Culture sequenced reads can be found under NCBI BioProject PRJNA1036577.
