# Supplementary material for: Giant viral signatures on the Greenland ice sheet
Source: Microbiome. 2024 May 17;12:91. doi: 10.1186/s40168-024-01796-y (PMC11100222; doi:10.1186/s40168-024-01796-y)
Supplement: Supplementary file 1 — Additional file 1. Figure S1-S7: Maximum-likelihood phylogenetic tree of the NCLDV core gene D5, RNAps, RNApl, MCP, mRNAc, A32, SFII, VLTF3, RNR. Sequences recovered from the environmental samples are presented in bold. Environmental sample types are specified in correspondence of each sequence. Branches are color-coded by order-level taxonomy. Dark dots at the nodes represent the bootstrap support value of >70. Figure S8: Normalized counts of the NCLDV marker genes by each assembly size for all the samples in this study. Analysis was carried out in 19 environmental metagenomes (MG) and 18 environmental metatranscriptomes (pooled) obtained from samples of cryoconite (n=1), ice core (n=3), green snow (n=2), red snow (n=5), dark ice (n=8), one metavirome (dark ice) and five snow algae culture genomic assemblies from the CCCryo collection. The ‘md’ (more depth) notation following select samples are those that were re-sequenced with higher metagenomic coverage. Symbols represent the sample types. Figure S9: Metagenomic SSU relative abundance. Sample descriptions can be found in Table 1 and Supplementary Table 1. The blank space above each bar is comprised of bacterial phyla. The ‘md’ (more depth) following a sample name marks those that were sequenced with a high average library coverage. The full abundance table can be found in Supplementary Table S10. Figure S10: Read mapping percentage transformed into log scale + 0.01 for comparable scales. Purple values are 0 reads mapped. Assemblies (left) and sample read files (right), MG are metagenomes, MG with ‘_2’ note the four samples that were sequenced with higher library coverage, and ‘MT’ are metatranscriptomes. The only culture to have reads recruit was Raphidonema_sempervirens_LIV13260. Sample types are labeled in the same way as Fig. 2. [file 40168_2024_1796_MOESM1_ESM.zip › Figure_S2a.pdf]

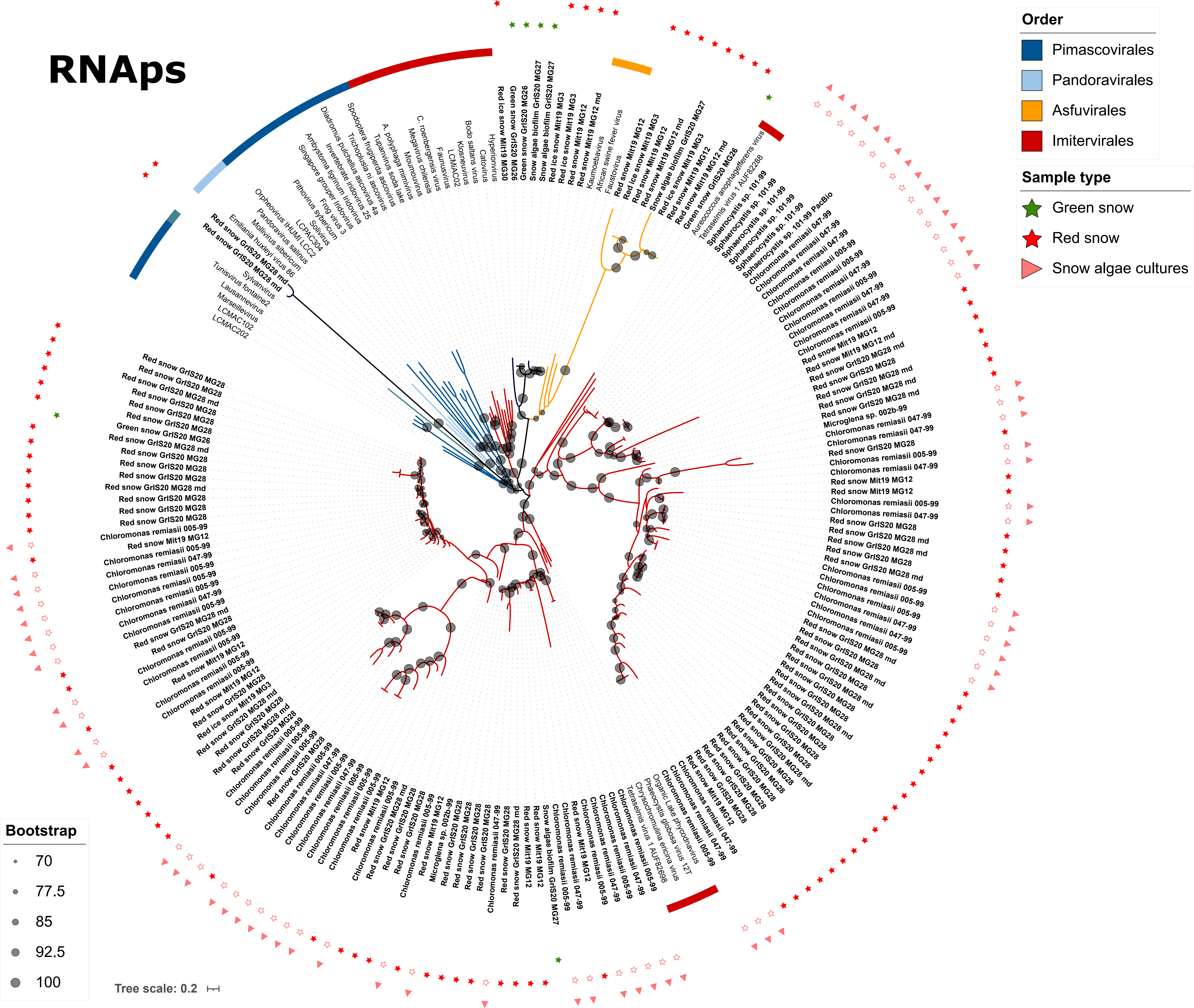

RNAps
Order
Pimascovirales
Pandoravirales
Asfuvirales
Imitervirales
Sample type
Green snow
Red snow
Snow algae cultures
Bootstrap
70
77.5
85
92.5
100
Tree scale: 0.2
