# Supplementary material for: Giant viral signatures on the Greenland ice sheet
Source: Microbiome. 2024 May 17;12:91. doi: 10.1186/s40168-024-01796-y (PMC11100222; doi:10.1186/s40168-024-01796-y)
Supplement: Supplementary file 1 — Additional file 1. Figure S1-S7: Maximum-likelihood phylogenetic tree of the NCLDV core gene D5, RNAps, RNApl, MCP, mRNAc, A32, SFII, VLTF3, RNR. Sequences recovered from the environmental samples are presented in bold. Environmental sample types are specified in correspondence of each sequence. Branches are color-coded by order-level taxonomy. Dark dots at the nodes represent the bootstrap support value of >70. Figure S8: Normalized counts of the NCLDV marker genes by each assembly size for all the samples in this study. Analysis was carried out in 19 environmental metagenomes (MG) and 18 environmental metatranscriptomes (pooled) obtained from samples of cryoconite (n=1), ice core (n=3), green snow (n=2), red snow (n=5), dark ice (n=8), one metavirome (dark ice) and five snow algae culture genomic assemblies from the CCCryo collection. The ‘md’ (more depth) notation following select samples are those that were re-sequenced with higher metagenomic coverage. Symbols represent the sample types. Figure S9: Metagenomic SSU relative abundance. Sample descriptions can be found in Table 1 and Supplementary Table 1. The blank space above each bar is comprised of bacterial phyla. The ‘md’ (more depth) following a sample name marks those that were sequenced with a high average library coverage. The full abundance table can be found in Supplementary Table S10. Figure S10: Read mapping percentage transformed into log scale + 0.01 for comparable scales. Purple values are 0 reads mapped. Assemblies (left) and sample read files (right), MG are metagenomes, MG with ‘_2’ note the four samples that were sequenced with higher library coverage, and ‘MT’ are metatranscriptomes. The only culture to have reads recruit was Raphidonema_sempervirens_LIV13260. Sample types are labeled in the same way as Fig. 2. [file 40168_2024_1796_MOESM1_ESM.zip › Figure_S6a.pdf]

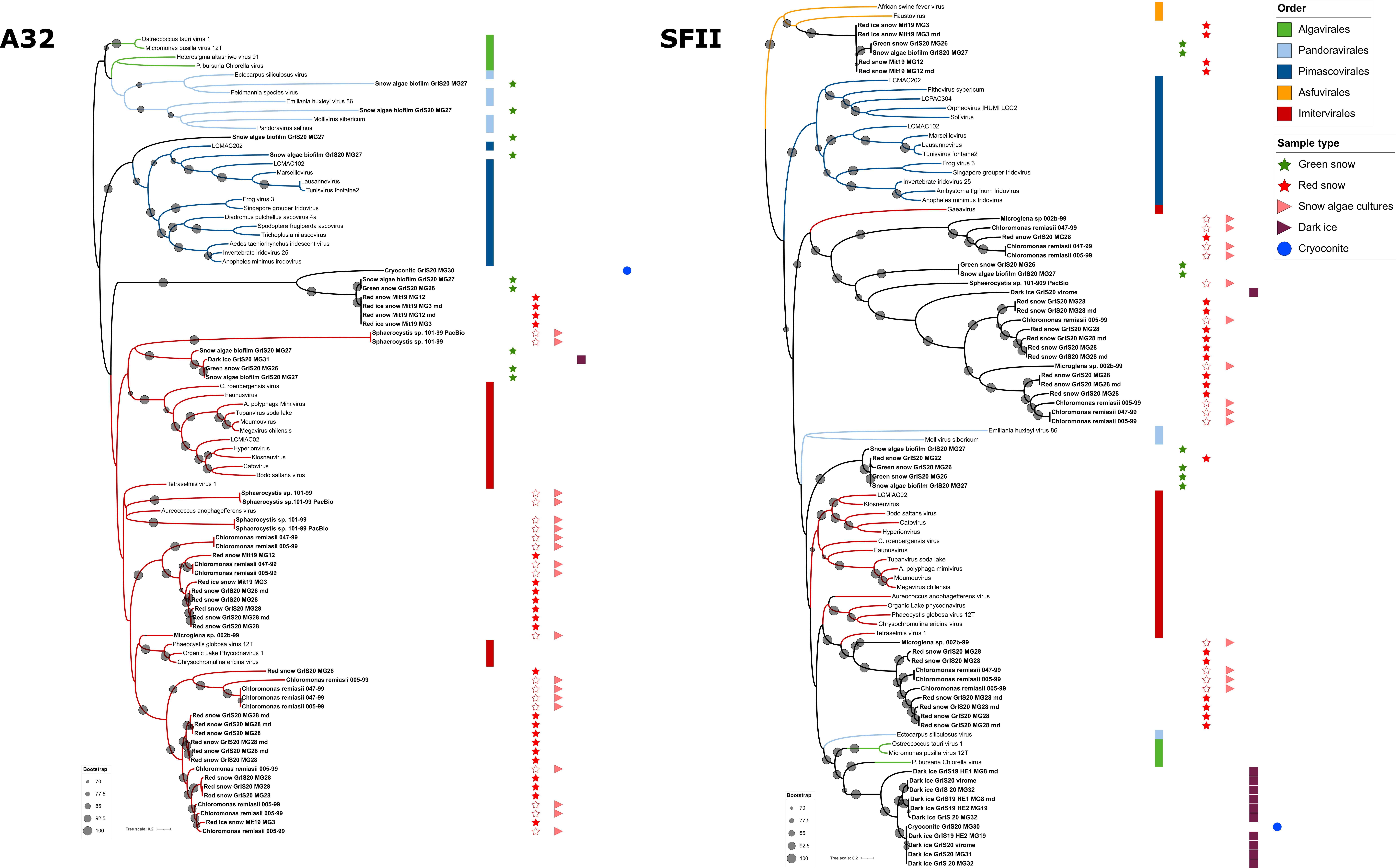
A32
Ostreococcus tauri virus 1
Micromonas pusilla virus 12T
Heterosigma akashiwo virus 01
P. bursaria Chlorella virus
Ectocarpus siliculosus virus
Snow algae biofilm GrIS20 MG27
Feldmannia species virus
Emiliania huxleyi virus 86
Snow algae biofilm GrIS20 MG27
Mollivirus sibericum
Pandoravirus salinus
Snow algae biofilm GrIS20 MG27
LCMAC202
Snow algae biofilm GrIS20 MG27
LCMAC102
Marseillevirus
Lausannevirus
Tunisvirus fontaine2
Frog virus 3
Singapore grouper Iridovirus
Diadromus pulchellus ascovirus 4a
Spodoptera frugiperda ascovirus
Trichoplusia ni ascovirus
Aedes taeniorhynchus iridescent virus
Invertebrate iridovirus 25
Anopheles minimus irodovirus
Cryoconite GrIS20 MG30
Snow algae biofilm GrIS20 MG27
Green snow GrIS20 MG26
Red snow Mit19 MG12
Red ice snow Mit19 MG3 md
Red snow Mit19 MG12 md
Red ice snow Mit19 MG3
Sphaerocystis sp. 101-99 PacBio
Sphaerocystis sp. 101-99
Snow algae biofilm GrIS20 MG27
Dark ice GrIS20 MG31
Green snow GrIS20 MG26
Snow algae biofilm GrIS20 MG27
C. roenbergensis virus
Faunusvirus
A. polyphaga Mimivirus
Tupanvirus soda lake
Moumouvirus
Megavirus chilensis
LCMiAC02
Hyperionvirus
Klosneuvirus
Catovirus
Bodo saltans virus
Tetraselmis virus 1
Sphaerocystis sp. 101-99
Sphaerocystis sp.101-99 PacBio
Aureococcus anophagefferens virus
Sphaerocystis sp. 101-99
Sphaerocystis sp. 101-99 PacBio
Chloromonas remiasii 047-99
Chloromonas remiasii 005-99
Red snow Mit19 MG12
Chloromonas remiasii 047-99
Chloromonas remiasii 005-99
Red ice snow Mit19 MG3
Red snow GrIS20 MG28 md
Red snow GrIS20 MG28
Red snow GrIS20 MG28
Red snow GrIS20 MG28 md
Red snow GrIS20 MG28
Microglena sp. 002b-99
Phaeocystis globosa virus 12T
Organic Lake Phycodnavirus 1
Chrysochromulina ericina virus
Red snow GrIS20 MG28
Chloromonas remiasii 005-99
Chloromonas remiasii 047-99
Chloromonas remiasii 047-99
Chloromonas remiasii 005-99
Red snow GrIS20 MG28 md
Red snow GrIS20 MG28 md
Red snow GrIS20 MG28
Red snow GrIS20 MG28 md
Red snow GrIS20 MG28 md
Red snow GrIS20 MG28
Chloromonas remiasii 005-99
Red snow GrIS20 MG28
Red snow GrIS20 MG28
Red snow GrIS20 MG28
Chloromonas remiasii 005-99
Chloromonas remiasii 005-99
Red ice snow Mit19 MG3
Chloromonas remiasii 005-99
Bootstrap
70
77.5
85
92.5
100
Tree scale: 0.2
SFII
African swine fever virus
Faustovirus
Red ice snow Mit19 MG3
Red ice snow Mit19 MG3 md
Green snow GrIS20 MG26
Snow algae biofilm GrIS20 MG27
Red snow Mit19 MG12
Red snow Mit19 MG12 md
LCMAC202
Pithovirus sybericum
LCPAC304
Orpheovirus IHUMI LCC2
Solivirus
LCMAC102
Marseillevirus
Lausannevirus
Tunisvirus fontaine2
Frog virus 3
Singapore grouper Iridovirus
Invertebrate iridovirus 25
Ambystoma tigrinum Iridovirus
Anopheles minimus Iridovirus
Gaeavirus
Microglena sp 002b-99
Chloromonas remiasii 047-99
Red snow GrIS20 MG28
Chloromonas remiasii 047-99
Chloromonas remiasii 005-99
Green snow GrIS20 MG26
Snow algae biofilm GrIS20 MG27
Sphaerocystis sp. 101-909 PacBio
Dark ice GrIS20 virome
Red snow GrIS20 MG28
Red snow GrIS20 MG28 md
Chloromonas remiasii 005-99
Red snow GrIS20 MG28
Red snow GrIS20 MG28 md
Red snow GrIS20 MG28
Red snow GrIS20 MG28 md
Microglena sp. 002b-99
Red snow GrIS20 MG28
Red snow GrIS20 MG28 md
Red snow GrIS20 MG28
Chloromonas remiasii 005-99
Chloromonas remiasii 047-99
Chloromonas remiasii 005-99
Emiliania huxleyi virus 86
Mollivirus sibericum
Snow algae biofilm GrIS20 MG27
Red snow GrIS20 MG22
Green snow GrIS20 MG26
Green snow GrIS20 MG26
Snow algae biofilm GrIS20 MG27
LCMiAC02
Klosneuvirus
Bodo saltans virus
Catovirus
Hyperionvirus
C. roenbergensis virus
Faunusvirus
Tupanvirus soda lake
A. polyphaga mimivirus
Moumouvirus
Megavirus chilensis
Aureococcus anophagefferens virus
Organic Lake phycodnavirus
Phaeocystis globosa virus 12T
Chrysochromulina ericina virus
Tetraselmis virus 1
Microglena sp. 002b-99
Red snow GrIS20 MG28
Red snow GrIS20 MG28
Chloromonas remiasii 047-99
Chloromonas remiasii 005-99
Chloromonas remiasii 005-99
Red snow GrIS20 MG28 md
Red snow GrIS20 MG28 md
Red snow GrIS20 MG28
Red snow GrIS20 MG28 md
Ectocarpus siliculosus virus
Ostreococcus tauri virus 1
Micromonas pusilla virus 12T
P. bursaria Chlorella virus
Dark ice GrIS19 HE1 MG8 md
Dark ice GrIS20 virome
Dark ice GrIS 20 MG32
Dark ice GrIS19 HE1 MG8 md
Dark ice GrIS19 HE2 MG19
Dark ice GrIS 20 MG32
Cryoconite GrIS20 MG30
Dark ice GrIS19 HE2 MG19
Dark ice GrIS20 virome
Dark ice GrIS20 MG31
Dark ice GrIS 20 MG32
Bootstrap
70
77.5
85
92.5
100
Tree scale: 0.2
Order
Algavirales
Pandoravirales
Pimascovirales
Asfuvirales
Imitervirales
Sample type
Green snow
Red snow
Snow algae cultures
Dark ice
Cryoconite
