# Supplementary material for: Giant viral signatures on the Greenland ice sheet
Source: Microbiome. 2024 May 17;12:91. doi: 10.1186/s40168-024-01796-y (PMC11100222; doi:10.1186/s40168-024-01796-y)
Supplement: Supplementary file 1 — Additional file 1. Figure S1-S7: Maximum-likelihood phylogenetic tree of the NCLDV core gene D5, RNAps, RNApl, MCP, mRNAc, A32, SFII, VLTF3, RNR. Sequences recovered from the environmental samples are presented in bold. Environmental sample types are specified in correspondence of each sequence. Branches are color-coded by order-level taxonomy. Dark dots at the nodes represent the bootstrap support value of >70. Figure S8: Normalized counts of the NCLDV marker genes by each assembly size for all the samples in this study. Analysis was carried out in 19 environmental metagenomes (MG) and 18 environmental metatranscriptomes (pooled) obtained from samples of cryoconite (n=1), ice core (n=3), green snow (n=2), red snow (n=5), dark ice (n=8), one metavirome (dark ice) and five snow algae culture genomic assemblies from the CCCryo collection. The ‘md’ (more depth) notation following select samples are those that were re-sequenced with higher metagenomic coverage. Symbols represent the sample types. Figure S9: Metagenomic SSU relative abundance. Sample descriptions can be found in Table 1 and Supplementary Table 1. The blank space above each bar is comprised of bacterial phyla. The ‘md’ (more depth) following a sample name marks those that were sequenced with a high average library coverage. The full abundance table can be found in Supplementary Table S10. Figure S10: Read mapping percentage transformed into log scale + 0.01 for comparable scales. Purple values are 0 reads mapped. Assemblies (left) and sample read files (right), MG are metagenomes, MG with ‘_2’ note the four samples that were sequenced with higher library coverage, and ‘MT’ are metatranscriptomes. The only culture to have reads recruit was Raphidonema_sempervirens_LIV13260. Sample types are labeled in the same way as Fig. 2. [file 40168_2024_1796_MOESM1_ESM.zip › Figure_S7a.pdf]

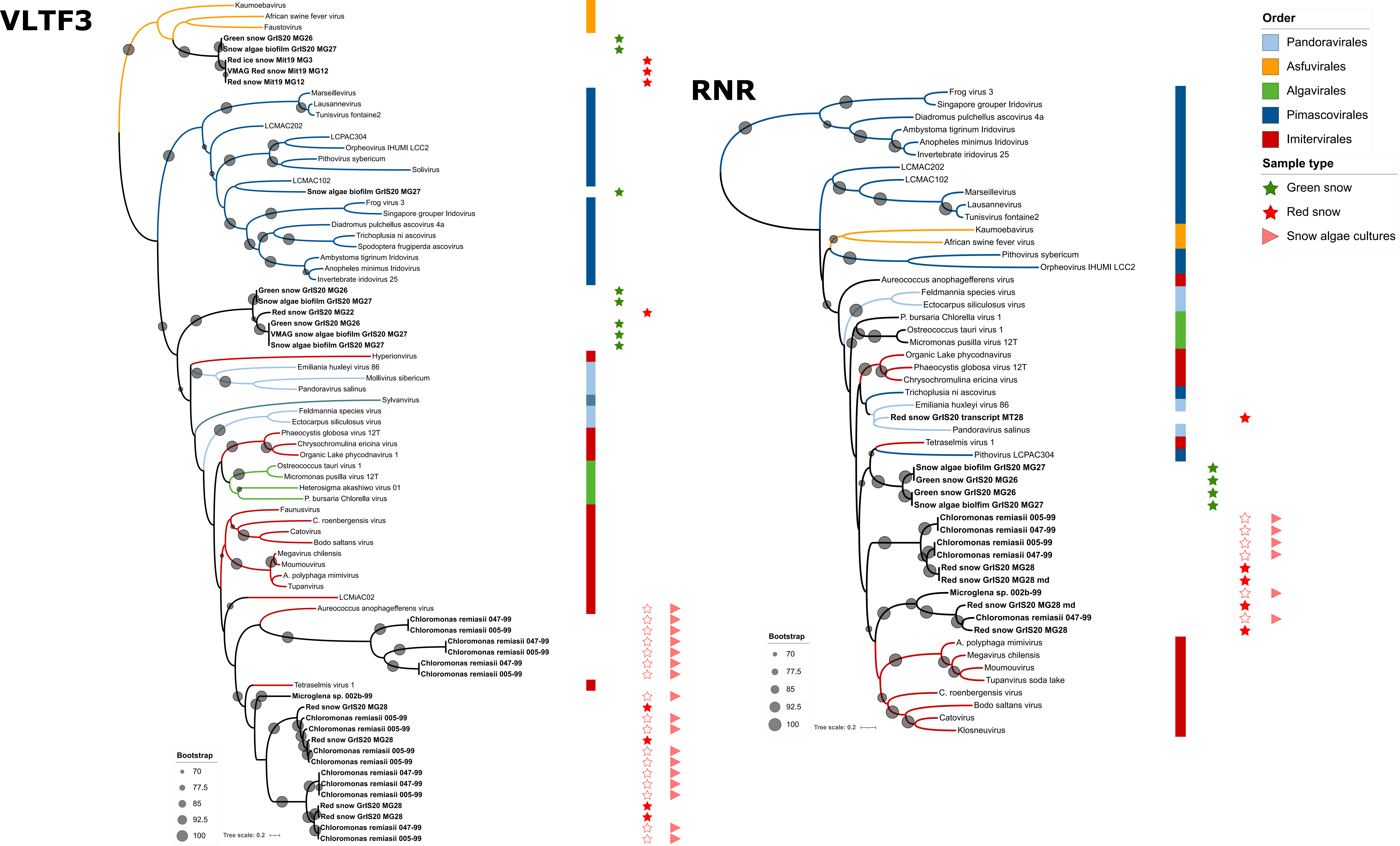
VLTF3
Kaumoebavirus
African swine fever virus
Faustovirus
Green snow GrIS20 MG26
Snow algae biofilm GrIS20 MG27
Red ice snow Mit19 MG3
VMAG Red snow Mit19 MG12
Red snow Mit19 MG12
Marseillevirus
Lausannevirus
Tunisvirus fontaine2
LCMAC202
LCPAC304
Orpheovirus IHUMI LCC2
Pithovirus sybericum
Solivirus
LCMAC102
Snow algae biofilm GrIS20 MG27
Frog virus 3
Singapore grouper Iridovirus
Diadromus pulchellus ascovirus 4a
Trichoplusia ni ascovirus
Spodoptera frugiperda ascovirus
Ambystoma tigrinum Iridovirus
Anopheles minimus Iridovirus
Invertebrate iridovirus 25
Green snow GrIS20 MG26
Snow algae biofilm GrIS20 MG27
Red snow GrIS20 MG22
Green snow GrIS20 MG26
VMAG snow algae biofilm GrIS20 MG27
Snow algae biofilm GrIS20 MG27
Hyperionvirus
Emiliania huxleyi virus 86
Mollivirus sibericum
Pandoravirus salinus
Sylvanvirus
Feldmannia species virus
Ectocarpus siliculosus virus
Phaeocystis globosa virus 12T
Chrysochromulina ericina virus
Organic Lake phycodnavirus 1
Ostreococcus tauri virus 1
Micromonas pusilla virus 12T
Heterosigma akashiwo virus 01
P. bursaria Chlorella virus
Faunusvirus
C. roenbergensis virus
Catovirus
Bodo saltans virus
Megavirus chilensis
Moumouvirus
A. polyphaga mimivirus
Tupanvirus
LCMiAC02
Aureococcus anophagefferens virus
Chloromonas remiasii 047-99
Chloromonas remiasii 005-99
Chloromonas remiasii 047-99
Chloromonas remiasii 005-99
Chloromonas remiasii 047-99
Chloromonas remiasii 005-99
Tetraselmis virus 1
Microglena sp. 002b-99
Red snow GrIS20 MG28
Chloromonas remiasii 005-99
Chloromonas remiasii 005-99
Red snow GrIS20 MG28
Chloromonas remiasii 005-99
Chloromonas remiasii 005-99
Chloromonas remiasii 047-99
Chloromonas remiasii 047-99
Chloromonas remiasii 005-99
Red snow GrIS20 MG28
Red snow GrIS20 MG28
Chloromonas remiasii 047-99
Chloromonas remiasii 005-99
Bootstrap
70
77.5
85
92.5
100
Tree scale: 0.2
RNR
Frog virus 3
Singapore grouper Iridovirus
Diadromus pulchellus ascovirus 4a
Ambystoma tigrinum Iridovirus
Anopheles minimus Iridovirus
Invertebrate iridovirus 25
LCMAC202
LCMAC102
Marseillevirus
Lausannevirus
Tunisvirus fontaine2
Kaumoebavirus
African swine fever virus
Pithovirus sybericum
Orpheovirus IHUMI LCC2
Aureococcus anophagefferens virus
Feldmannia species virus
Ectocarpus siliculosus virus
P. bursaria Chlorella virus 1
Ostreococcus tauri virus 1
Micromonas pusilla virus 12T
Organic Lake phycodnavirus
Phaeocystis globosa virus 12T
Chrysochromulina ericina virus
Trichoplusia ni ascovirus
Emiliania huxleyi virus 86
Red snow GrIS20 transcript MT28
Pandoravirus salinus
Tetraselmis virus 1
Pithovirus LCPAC304
Snow algae biofilm GrIS20 MG27
Green snow GrIS20 MG26
Green snow GrIS20 MG26
Snow algae biolfim GrIS20 MG27
Chloromonas remiasii 005-99
Chloromonas remiasii 047-99
Chloromonas remiasii 005-99
Chloromonas remiasii 047-99
Red snow GrIS20 MG28
Red snow GrIS20 MG28 md
Microglena sp. 002b-99
Red snow GrIS20 MG28 md
Chloromonas remiasii 047-99
Red snow GrIS20 MG28
A. polyphaga mimivirus
Megavirus chilensis
Moumouvirus
Tupanvirus soda lake
C. roenbergensis virus
Bodo saltans virus
Catovirus
Klosneuvirus
Bootstrap
70
77.5
85
92.5
100
Tree scale: 0.2
Order
Pandoravirales
Asfuvirales
Algavirales
Pimascovirales
Imitervirales
Sample type
Green snow
Red snow
Snow algae cultures
